# Disease extent and anti‐tubercular treatment response correlates with *Mycobacterium tuberculosis*‐specific CD4 T‐cell phenotype regardless of HIV‐1 status

**DOI:** 10.1002/cti2.1176

**Published:** 2020-09-28

**Authors:** Catherine Riou, Elsa Du Bruyn, Sheena Ruzive, Rene T Goliath, Cecilia S Lindestam Arlehamn, Alessandro Sette, Alan Sher, Daniel L Barber, Robert J Wilkinson

**Affiliations:** ^1^ Wellcome Centre for Infectious Disease Research in Africa Institute of Infectious Disease and Molecular Medicine University of Cape Town Observatory South Africa; ^2^ Division of Immunology Department of Pathology University of Cape Town Observatory South Africa; ^3^ Division of Vaccine Discovery La Jolla Institute for Immunology La Jolla CA USA; ^4^ Department of Medicine University of California San Diego La Jolla CA USA; ^5^ Immunobiology Section Laboratory of Parasitic Diseases National Institute of Allergy and Infectious Diseases National Institutes of Health Bethesda MD USA; ^6^ T Lymphocyte Biology Section Laboratory of Parasitic Diseases National Institute of Allergy and Infectious Diseases National Institutes of Health Bethesda MD USA; ^7^ Department of Infectious Diseases Imperial College London London UK; ^8^ Department of Medicine University of Cape Town Observatory South Africa; ^9^ The Francis Crick Institute London UK

**Keywords:** tuberculosis, CD4 response, disease severity, treatment response

## Abstract

**Objectives:**

The development of non‐sputum‐based assays for tuberculosis (TB) diagnosis and treatment monitoring is a key priority. Recent data indicate that whole blood‐based assays to assess the phenotype of *Mycobacterium tuberculosis* (Mtb)‐specific CD4 T cells hold promise for this purpose and require further investigation in well‐characterised TB cohorts. In this study, we investigated the relationship between the phenotypic signature of Mtb‐specific CD4 responses, TB disease extent and treatment response.

**Methods:**

Using flow cytometry, we measured the expression of phenotypic and functional markers (HLA‐DR, CD27, CD153, KLRG1, IL‐2, MIP‐1β, TNF‐α and IFN‐γ) on Mtb‐specific CD4 T‐cells in whole blood from 161 participants of varying TB and HIV status. TB disease extent was graded as a continuum using the Xpert_ct_ value, C‐reactive protein, Timika radiographic score and monocyte/lymphocyte ratio.

**Results:**

The phenotypic profile of Mtb‐specific CD4 T cells pre‐anti‐tubercular treatment (ATT) strongly correlated with disease extent, irrespective of HIV status. ATT associated with major changes in the phenotype of Mtb‐specific CD4 T cells, with decreased expression of HLA‐DR and increased CD27 and CD153 expression. Principal component analysis showed an almost complete separation between latent TB infection (LTBI) and active TB (aTB) pre‐ATT groups, whereas the profile of the aTB post‐ATT group overlapped with the LTBI group. However, in patients experiencing treatment failure or relapse, no significant changes were observed in Mtb‐specific CD4 T‐cell phenotype pre‐ and post‐ATT.

**Conclusion:**

Whole blood‐based assays of Mtb‐specific CD4 T‐cell activation and maturation markers can be used as non‐sputum‐based biomarkers of disease extent and treatment monitoring in TB, regardless of HIV‐1 status.

## Graphical abstract

A better understanding of the relationship between host immune responses, tuberculosis (TB) disease severity and treatment outcome is key to the development of novel TB diagnostic and treatment monitoring tools. In this study, we defined the profile of *Mycobacterium tuberculosis*‐specific IFNγ^+^ CD4 T‐cell response in TB patients, analysed its evolution after completion of a standard anti‐tubercular treatment regimen and evaluated how the host CD4 response associates with clinical TB extent and treatment response.

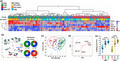

## Introduction

Globally, 10 million people were diagnosed with tuberculosis (TB) and 1.45 million people died as a result of this infection in 2018.^1^ TB remains the leading cause of death in HIV‐1‐infected people where sputum smear‐negative, extra‐pulmonary disease is frequent and represents a particular challenge in diagnosis and treatment monitoring.^2^ Although significant strides have recently been made in TB vaccine development,^3^, ^4^ there is still no licensed vaccine to prevent TB in adults. The treatment for drug susceptible TB consists of a 6‐month long regimen of drugs that can cause adverse effects and drug–drug interactions, particularly in the context of concomitant antiretroviral therapy (ART).^5^ Thus, the development of new shortened TB regimens has been defined as a major goal by WHO^6^ to improve patient adherence, reduce transmission and reduce costs to patients and health systems. Different approaches are being assessed in ongoing clinical trials including increased doses of existing drugs, adjunctive host‐directed therapies and the use of new and repurposed drugs in anti‐TB treatment (ATT) regimens.^7^, ^8^ Sputum culture for *Mycobacterium tuberculosis* (Mtb), the current standard measure of monitoring treatment response, has a long turnaround time taking up to 42 days to yield results, and PCR‐based methods such as GeneXpert are not recommended to monitor treatment response owing to detection of DNA from dead bacteria.^9^ Thus, there is an urgent need for novel biomarkers that assess clinical treatment response both accurately and more rapidly than conventional methods such as sputum culture to aid clinical decision‐making in the programmatic setting and assist with the monitoring of ATT response in clinical trials.

There is evidence from studies using PET‐CT imaging that some TB patients may exhibit ongoing lung inflammation, suggestive of active TB (aTB) disease, accompanied by detectable Mtb mRNA in sputum and bronchoalveolar lavage samples after completing 6 months of ATT and up to 1 year of follow‐up.^10^ Thus, while may be possible to shorten ATT regimens for some, it may be necessary to prolong ATT in others to prevent relapse or recurrence of drug sensitive TB. A recent large population‐based cohort in South Africa showed high TB recurrence rates,^11^ in line with previous reports from high prevalence settings.^12^, ^13^ The cause of high rates of TB recurrence is likely to be multifactorial;^11^ however, strategies are required to identify and track those at highest risk. It has been shown that CD4 T‐cell activation status denoted by the expression of the MHC class II cell surface receptor HLA‐DR is indicative of TB risk and could potentially be investigated for this purpose.^14^ Furthermore, several studies have highlighted the potential of blood‐based assays as diagnostic tools; and while measuring the magnitude of Mtb‐specific T‐cell response does not offer much value for TB diagnostic^15^, ^16^ or treatment monitoring,^17^ assessing the phenotypic profile of these cells showed promising results. Indeed, the activation, memory differentiation or functional profile of Mtb‐specific CD4 T cells has been shown to relate to TB disease activity^15^, ^18^, ^19^, ^20^, ^21^, ^22^, ^23^, ^24^, ^25^, ^26^, ^27^ and evaluate treatment response.^18^, ^19^, ^28^, ^29^ However, very few of these studies have included HIV‐infected participants. Thus, in this study, we set out to better understand the relationship between the phenotypic attributes of Mtb‐specific CD4 T cells, TB disease severity and treatment response. We comprehensively investigated the expression of multiple promising phenotypic markers on Mtb‐specific CD4 T cells using flow cytometry measured in whole blood samples from a well‐characterised cohort of HIV‐infected and HIV‐uninfected persons with latent TB infection (LTBI) and aTB disease before and after ATT completion.

## Results

### Study population

The clinical characteristics of participants are presented in Table 1. Participants (*n* = 161) were classified into four groups according to their HIV‐1 and TB status: LTBI/HIV^−^ (*n* = 35), LTBI/HIV^+^ (*n* = 31), aTB/HIV^−^ (*n* = 41), and aTB/HIV^+^ (*n* = 54). Median age was comparable between the four groups. IGRA values were comparable between the HIV‐uninfected and HIV‐infected LTBI groups (*P* = 0.14). The Xpert MTB/RIF cycle threshold (Xpert_ct_) was higher in HIV‐infected than in HIV‐uninfected patients (median: 23.3 vs 18.5, respectively, *P* = 0.001). HIV infection status did not significantly affect the level of plasma C‐reactive protein (CRP) in aTB patients (medians: 72 µg mL^−1^ in HIV^+^ and 100 µg mL^−1^ in HIV^−^, *P* = 0.99). Finally, HIV‐infected participants with LTBI had a significantly lower plasma HIV‐1 viral load (VL) and higher absolute CD4 count than the HIV‐infected aTB group (median VL: < 20 vs 43 415 copies mL^−1^, *P* = 0.0003 and median CD4: 490 vs 206 cells mm^−3^, *P* < 0.0001, respectively). These differences are due to higher ART usage in the LTBI group than in the aTB group (80.6% vs 38.9%, respectively, *P* = 0.0002). Follow‐up blood samples were available for 28 HIV‐uninfected participants and 33 for HIV‐infected patients after the completion of standard ATT regimen at week 24.

**Table 1:** Clinical characteristics of study participants

	Baseline				Week 24 (paired)		*P*‐values
	LTBI/HIV^−^	LTBI/HIV^+^	aTB/HIV^−^	aTB/HIV^+^	Post‐ATT HIV^−^	Post‐ATT HIV^+^	
*N*	**35**	**31**	**41**	**54**	**28**	**33**	
Age (years)^†^	**31** (23–38)	**34** (32–41)	**30** (24–43)	**37** (32–45)	**30** (23–42)	**37** (31–45)	
Gender (F/M)	19/16	23/8	10/31	17/37	6/22	10/23	
IGRA (IU mL^−1^)^†^	**6.6** (2.4–10)	**3.2** (1.9–8.4)	nd	nd	nd	nd	0.14
CD4 (cells mm^−3^)^†^	nd	**490** (380–702)	nd	**206** (121–355)	nd	**355** (184–456)	**< 0.0001** ^‡^/**0.01** ^§^
VL (mRNA copies mL^−1^)^†^	na	**< 20** (< 20–17 514)	na	**43 415** (157–127 940)	na	**< 20** (< 20–1620)	**0.0003** ^‡^/**< 0.0001** ^§^
On ART (%)	na	**80.6**	na	**38.9**	na	**100**	**0.0002** ^‡^/**< 0.0001** ^§^
Xpert_ct_ value^†^	na	na	**18.5** (15.7–21.8)	**23.3** (18.9–27.3)	nd	nd	**0.001**
TTP (days)†	na	na	**7** (6–9)	**10** (8–15)	nd	nd	**0.003**
Timika score†	na	na	**58.7** (28.9–99.2)	**30.8** (6.7–64.3)	**12.5** (6.1–33.3)	**10** (5–39.1)	**0.011** ^¶^/0.34^#^
Mono/Lympho ratio†	**0.39** (0.22–0.57)	**0.24** (0.17–0.28)	**0.54** (0.31–0.73)	**0.39** (0.22–0.57)	nd	nd	0.12^#^/0.05^¶^
CRP (µg mL^−1^)^†^	**1** (1–4)	**3** (2–10)	**100** (27.5–115)	**72** (36–123)	nd	nd	**0.011** ^¶^/0.86^¶^
Analysed by flow (*n*)	35	31	33	37	28	33	
Mtb response > 30 events (*n*)	28	31	31	35	26	29	

ART, antiretroviral treatment; aTB, active tuberculosis disease; ATT, anti‐tubercular treatment; CRP, C‐reactive protein; F, female; IGRA, Interferon Gamma Release Assay; LTBI, latent tuberculosis infection; M, male; na, not applicable; nd, not done; VL, HIV‐1 viral load; Xpert_ct_, Xpert MTB/RIF cycle threshold.

*P*‐values were calculated using a non‐parametric Mann–Whitney test for all measurements except for ART usage where a chi‐square test was applied. For IGRA, results are reported as (TB Ag‐Nil). The cut‐off point for positivity was 0.36 IU mL^−1^. The Xpert cycle threshold value for each participant was defined as the average value of the 5 probes used in the assay. Clinical data (including Xpert_ct_, Timika score or CRP) were missing for six aTB participants. Bold font has been used to highlight medians and significant *P‐*values.

^†^ Median and interquartile ranges.

^‡^ *P*‐value between the LTBI/HIV^−^ and aTB/HIV^+^ groups.

^§^ *P*‐value between the aTB/baseline and aTB/Week 24.

^¶^ *P*‐value between the aTB/HIV^−^ and aTB/HIV^+^ groups.

^#^ *P*‐value between the Week 24/HIV^−^ and Week 24/HIV^+^.

^††^ *P*‐value between the LTBI/HIV^−^ and LTBI/HIV^+^ groups.

### Measures of TB disease extent

Five different measures of TB disease extent were assessed at the time of ATT initiation: sputum Xpert_ct_ values, sputum Mtb culture time to positivity (TTP), Timika radiographic severity score, serum CRP levels and peripheral blood monocyte to lymphocyte ratio (M/L ratio). Xpert_ct_ values and culture TTP were significantly lower in HIV‐uninfected patients than in those infected with HIV (*P* = 0.001 and *P* = 0.003, respectively), while the Timika score was significantly higher in HIV‐uninfected participants (*P* = 0.011, Supplementary figure 1a). This can be ascribed to the fact that cavitatory disease is more frequent in HIV‐uninfected persons. In the aTB group, no differences were observed in plasma CRP concentration or the M/L ratio between HIV‐infected and HIV‐uninfected participants. However, CRP and the M/L ratio were higher in those with aTB than those with LTBI, regardless of HIV status (*P* < 0.0001 for all sub‐group comparisons, Supplementary figure 1a). All measures of disease extent were assessed for the strength of their correlation with each other by Spearman correlation rank (*r*) values and further stratified by HIV status (Supplementary figure 1a and c). There was significant correlation between all the measures regardless of HIV status, with Xpert_ct_ value vs culture TTP, Xpert_ct_ value vs Timika score and CRP vs M/L ratio showing the strongest correlations overall (*P* < 0.0001).

**Figure 1:**
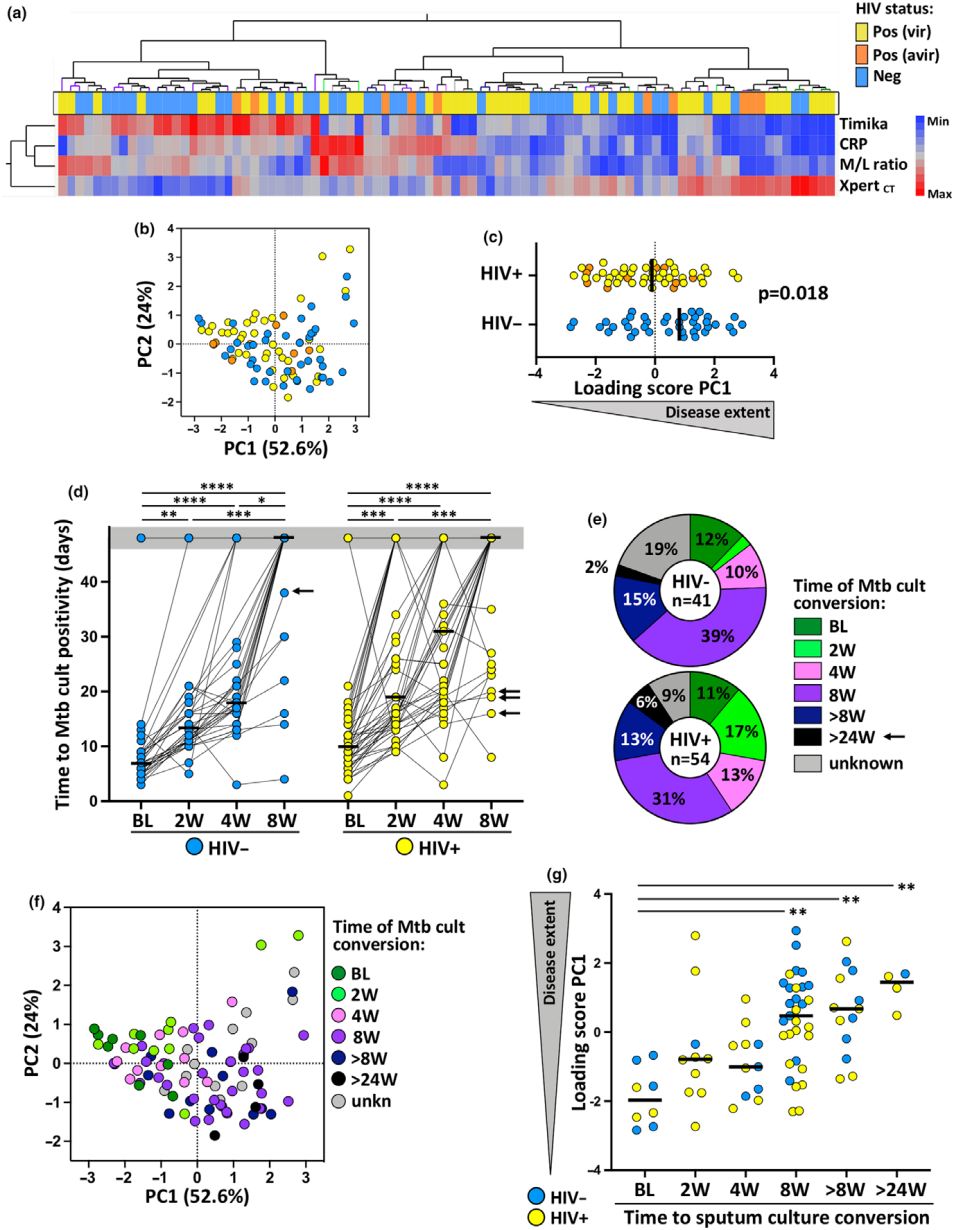
Delineation of tuberculosis (TB) disease score based on bacterial burden, systemic inflammation and lung damage and its relationship to treatment outcome. **(a)** A non‐supervised two‐way hierarchical cluster analysis (Ward method) was employed to grade TB disease, using the Timika score, plasma C‐reactive protein concentration, monocyte/lymphocyte ratio (M/L ratio) and Xpert_ct_ value. The HIV status of each patient is indicated at the bottom of the dendrogram, HIV‐uninfected (Neg, blue, *n* = 40), viraemic HIV‐infected persons (Pos vir, yellow, *n* = 10) and aviraemic HIV‐infected persons (Pos avir, orange, *n* = 39). **(b)** Principal component analysis on correlations, based on the four clinical parameters, was used to explain the variance of the data distribution in the cohort. Each dot represents a participant and is colour‐coded according to HIV status. The two axes represent principal components 1 (PC1) and 2 (PC2). Their contribution to the total data variance is shown as a percentage. PC3 contributed 12.4% of total variance and is not shown. **(c)** PC1 score values extracted from (b) were compared between HIV‐infected and HIV‐uninfected active TB (aTB) patients. Bars represent medians, and statistical comparison was performed using non‐parametric Mann–Whitney test. **(d)** Evolution of the time to Mtb culture positivity measured in sputum samples from baseline to 8 weeks post‐anti‐tubercular treatment initiation in HIV‐uninfected (blue) and HIV‐infected (yellow) patients. Bars indicate median. Statistical comparisons were defined using a Kruskal–Wallis test, adjusted for multiple comparisons (Dunn's test). Arrows indicate patients who were still sputum Mtb culture positive at week 24. **(e)** Distribution of participants according to time to Mtb culture conversion in sputum. Unknown corresponds to individuals for whom sputum Mtb culture results were not available at week 8. Those who were culture negative at baseline and were diagnosed with aTB using sputum Xpert in addition to clinical evaluation (see Supplementary table 1) are denoted as ‘BL’ sputum converters. **(f)** Principal component analysis on correlations, based on the four clinical parameters. Each dot represents a participant and is colour‐coded according to Time to Mtb culture conversion. **(g)** Relationship between PC1 score values and time to Mtb culture conversion. Bars represent median. Statistical comparisons were defined using a Kruskal–Wallis test, adjusted for multiple comparisons (Dunn's test).

Hierarchical cluster analysis of markers of disease extent revealed two major subgroups of aTB patients: cluster 1 being associated with a phenotype associated with evidence of greater pulmonary and systemic disease extent (lower Xpert_ct_ values and higher CRP, monocyte to lymphocyte ratio and Timika score) and dominated by HIV‐uninfected patients (57%), whereas the other phenotype (cluster 2), characterised by lower sputum bacterial load, systemic inflammation and radiographic disease extent (higher Xpert_ct_ values and lower CRP, M/L ratio and Timika score), was dominated by HIV‐infected patients (63%) (Figure 1a). Furthermore, principal component analysis showed that the principal component 1 (PC1) accounted for 52.6% of the observed variance in measures of disease severity in the aTB cohort (Figure 1b), with significantly higher PC1 loading scores being observed in HIV‐uninfected than in HIV‐infected participants (*P* = 0.018, Figure 1c). Sputum culture TTP, a variable not included in the hierarchical cluster and principal component analysis (PCA) to avoid collinearity with sputum Xpert_ct_ values, progressively prolonged with duration of ATT regardless of HIV‐1 status, with most participants showing culture conversion by week 8 of treatment (Figure 1d and e). In keeping with the decline in sputum bacterial load, there was a significant decline in plasma CRP concentration, M/L ratio and Timika score with increasing duration of ATT in both HIV‐infected and HIV‐uninfected patients (Supplementary figure 2). Those with higher PC1 loading scores (e.g. greater pulmonary and systemic disease extent) at baseline tended to show slower bacterial clearance with later conversion time of their sputum culture during the course of ATT (≥ 8 weeks) compared to patients exhibiting low PC1 loading scores (Figure 1f and g).

### The impact of ATT on the magnitude and polyfunctional profile of the Mtb‐specific CD4 T‐cell response

**Figure 2:**
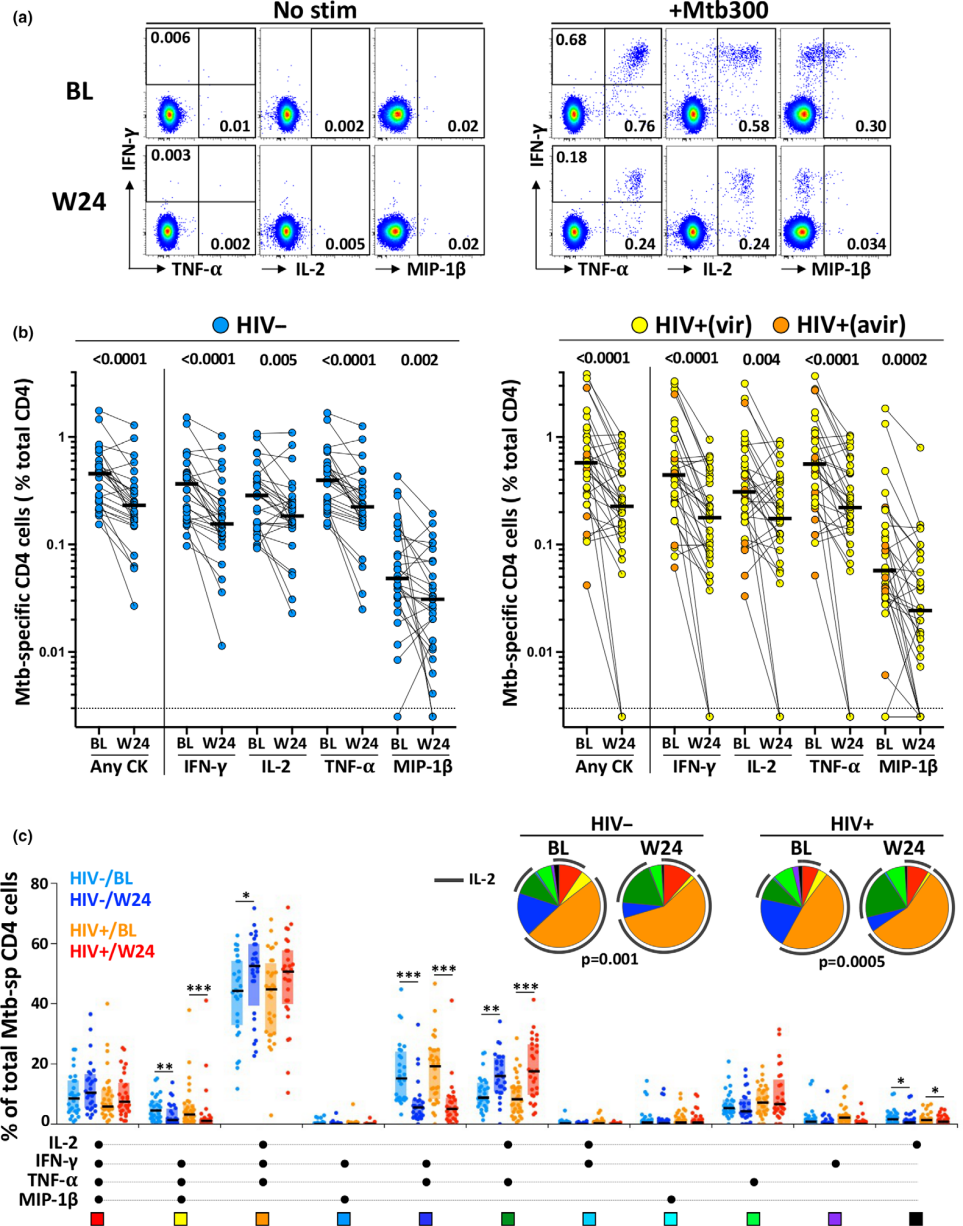
Comparison of the magnitude and polyfunctional profile of Mtb‐specific CD4 T‐cell responses at baseline and 24 weeks post‐anti‐tubercular treatment (ATT) initiation. **(a)** Representative flow plots of IFN‐γ, TNF‐α, IL‐2 and MIP‐1β expression in CD4 T cells in whole blood stimulated with a Mtb peptide pool (+Mtb300) or unstimulated (No stim). Baseline (BL) is shown on top and 24 weeks post‐ATT (W24) at the bottom. Number represents cytokine‐positive cells expressed as a percentage of total CD4 T cells. **(b)** Magnitude of Mtb‐specific CD4 T cells at BL and W24 in HIV‐uninfected (left, *n* = 28) and HIV‐infected (right, *n* = 33) patients. Bars represent medians. Statistical comparisons were defined using a non‐parametric Mann–Whitney test. **(c)** The polyfunctional profile of Mtb300‐specific CD4 T cells pre‐ (BL) and 24 weeks post‐ATT (W24) in HIV‐uninfected and HIV‐infected participants. The *x*‐axis displays each of the different response patterns, the composition of which is denoted with a dot for the presence of IL‐2, IFN‐γ, TNF‐α and MIP‐1β. Only response patterns contributing more than 1% to the overall Mtb responses are depicted. The proportion of each response pattern contributing to the total Mtb300‐specific CD4 response per individual is shown. The median (black bar) and interquartile ranges (box) are shown. Each response pattern is colour‐coded, and data are summarised in the pie charts, where each pie slice represents the median contribution of each response pattern to the total Mtb300 response. A Wilcoxon rank test was used to compare response patterns between groups (***P < 0.001, **P < 0.01, *P < 0.05). Statistical differences between pie charts were defined using a permutation test.

First, we compared the frequency of the CD4 T‐cell response to a pool of 300 Mtb‐derived peptides (Mtb300) in the aTB group at baseline and at the end of ATT (week 24). At baseline, the overall frequency of Mtb‐specific CD4 T cells was comparable in HIV‐uninfected and HIV‐infected patients (medians: 0.47% and 0.57%, respectively, *P* = 0.15). By week 24 of ATT, there was a median reduction of 36% in the overall frequency of the Mtb‐specific CD4 T‐cell response in those who were HIV‐uninfected, and a median reduction of 59% in HIV‐infected persons (*P* < 0.0001, Figure 2a and b). Due to concomitant ART usage in HIV‐infected patients, we took into account the variation in absolute CD4^+^ T‐cell counts between baseline and W24, and the absolute number of Mtb‐specific CD4^+^ T cells was compared between the two time points. Despite a significant increase in CD4 count in HIV‐infected patients initiating ART (medians: 226 cells mm^−3^ at BL vs 335 cells mm^−3^ at W24, *P* = 0.014), the absolute number of Mtb‐specific CD4 T cells remained approximately twofold lower at W24 compared to BL (median: 1.39 cells mm^−3^ vs 0.75 cells mm^−3^, respectively; *P* = 0.001, Supplementary figure 3a and b). This contraction of Mtb‐specific CD4 T cells could be related to the elimination of the activated effector antigen‐specific T cells upon pathogen clearance. Moreover, in the context of HIV infection, suboptimal immune reconstitution, despite long‐term ART and viral suppression, has been reported.^30^ Thus, incomplete restoration of pathogen‐specific T cells could also contribute to the more rapid contraction of Mtb‐specific CD4 T cells observed in the HIV‐infected group after ATT.

Furthermore, we quantified the polyfunctional potential of Mtb300‐specific CD4 T cells based on their capacity to co‐express IL‐2, IFN‐γ, TNF‐α or MIP‐1β (Figure 2c). While no significant difference was observed in the polyfunctional profile of Mtb300‐specific CD4 T cells at baseline between HIV‐uninfected and HIV‐infected patients, completion of ATT associated with a significant change in the overall functional capacity of Mtb‐ responding CD4 cells in both groups (*P* = 0.001 and *P* = 0.0005, respectively). This was characterised by the contraction of IFN‐γ, TNF‐α double‐positive cells and IFN‐γ, TNF‐α, MIP1β co‐producing cells and counterbalanced by the expansion of IL‐2^+^ TNFα^+^ CD4 cells. Overall, ATT resulted in similar changes in the HIV‐uninfected and HIV‐infected groups at week 24, characterised by the expansion of IL‐2 producing CD4 T cells, suggesting that ATT could improve cell proliferation capacity as previously described.^31^

### Phenotypic signature of Mtb‐specific IFNγ^+^ CD4 T cells in LTBI and aTB at BL and week 24

**Figure 3:**
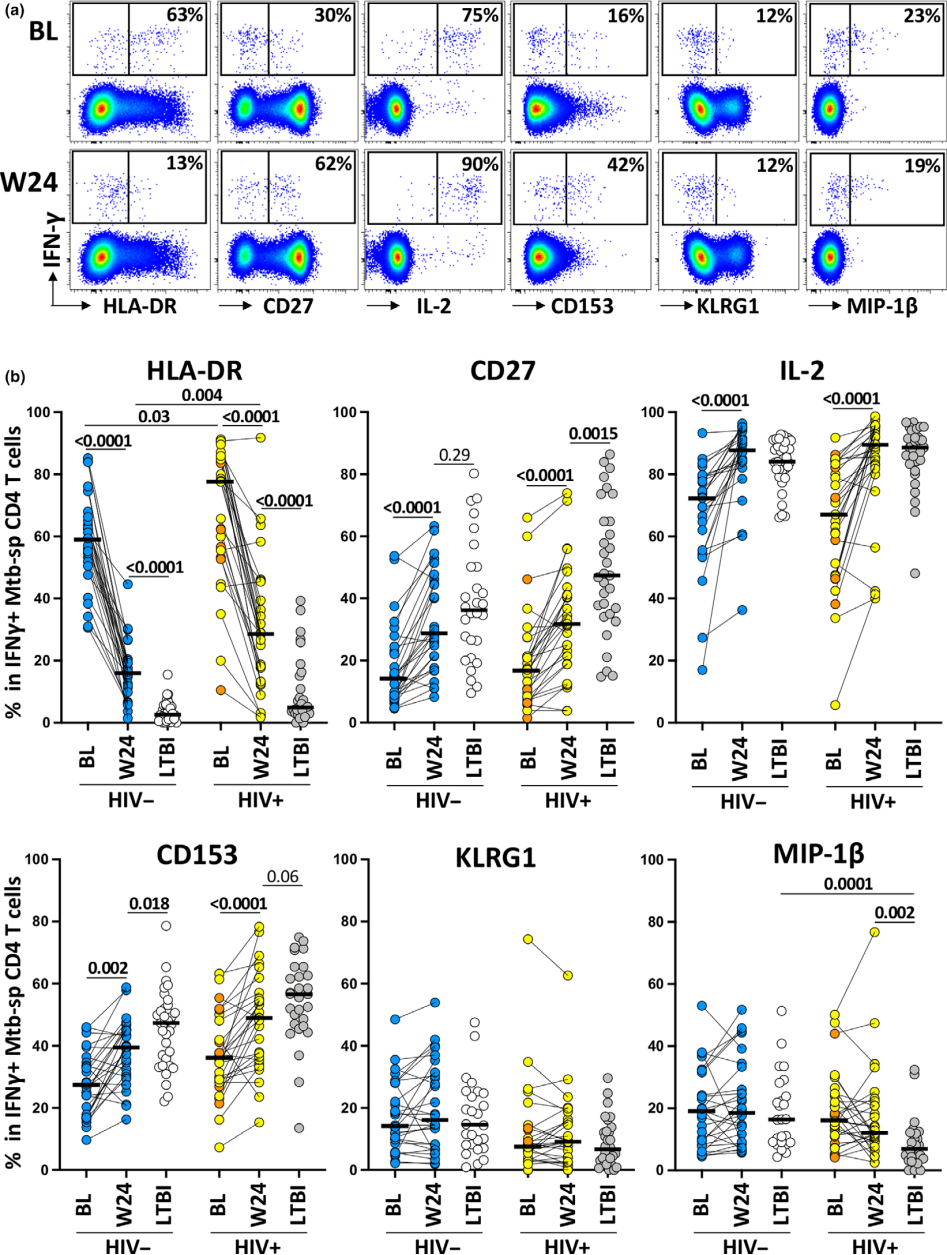
Comparison of the phenotypic profile of Mtb‐specific IFNγ^+^ CD4 T cells in active tuberculosis (aTB) patients (pre‐ and post‐anti‐tubercular treatment [ATT]) and in latent tuberculosis infection (LTBI) participants. **(a)** Representative flow plots of HLA‐DR, CD27, IL‐2, CD153, KLRG1 and MIP‐1β expression by IFNγ^+^ CD4 T cells in one aTB patients at baseline (BL, top) and 24 weeks post‐ATT initiation (W24, bottom). The number represents the percentage of cells positive for each marker within the IFNγ^+^ CD4 T cells. **(b)** Summary graphs of the expression of each studied marker by IFNγ^+^ CD4 T cells. aTB/HIV^−^ are depicted in blue (*n* = 26), aTB/HIV^+^ (viraemic) in yellow (*n* = 9), aTB/HIV^+^ (aviraemic) in orange (*n* = 20), LTBI/HIV^−^ in white (*n* = 28) and LTBI/HIV^+^ in grey (*n* = 31). Only paired samples were included. Bars represent medians. Statistical comparisons were performed using a Wilcoxon rank test for paired samples or a Mann–Whitney test for unpaired samples.

To better understand the phenotypic attributes of Mtb‐specific CD4 T cells that associate with TB disease extent, we compared the expression of HLA‐DR (activation marker), CD27 (memory marker), IL‐2 (functional potential), CD153 (TNF superfamily member, implicated in Mtb protection), KLRG1 (co‐inhibitory molecule) and MIP‐1β (chemokine, CCR5 agonist) in the LTBI group and the aTB cohort (at baseline and week 24; Figure 3a and b). The frequency of HLA‐DR expressing Mtb300‐specific IFNγ^+^ CD4 T cells significantly declined between baseline and week 24 (*P* < 0.0001), although frequencies at week 24 remained significantly higher than that observed in the LTBI groups (*P* < 0.0001), regardless of HIV status. Conversely, frequencies of CD27, IL‐2 and CD153 expressing Mtb300‐specific IFNγ^+^ CD4 T cells significantly increased after ATT in both HIV‐infected and HIV‐uninfected persons. In the HIV‐uninfected aTB group, CD27 expression after ATT was comparable to that of the LTBI group. Interestingly, this was not the case for HIV‐infected individuals, where significantly lower expression of CD27 was seen at week 24 than for the LTBI group (*P* = 0.0015). This could be indicative of failure of the HIV co‐infected subgroup to reconstitute the pool of central memory CD4 T cells after ATT. CD153 expression on Mtb300‐specific IFNγ^+^ CD4 T cells was also only partially restored after ATT in HIV‐uninfected persons, with a similar trend seen in HIV‐infected patients. Additionally, no significant changes were observed in the expression of KLRG1 and MIP‐1β in Mtb300‐specific IFNγ^+^ CD4 T cells pre‐ and post‐ATT in both groups. Overall, these results concur with previous reports showing that the phenotypic profile of Mtb‐specific IFNγ^+^ CD4 T cells in aTB disease is associated with a highly differentiated and activated profile.^15^, ^18^, ^29^ However, the Mtb‐specific IFNγ^+^ CD4 T‐cell phenotypic profile was only partially restored after 24 weeks of ATT.

**Figure 4:**
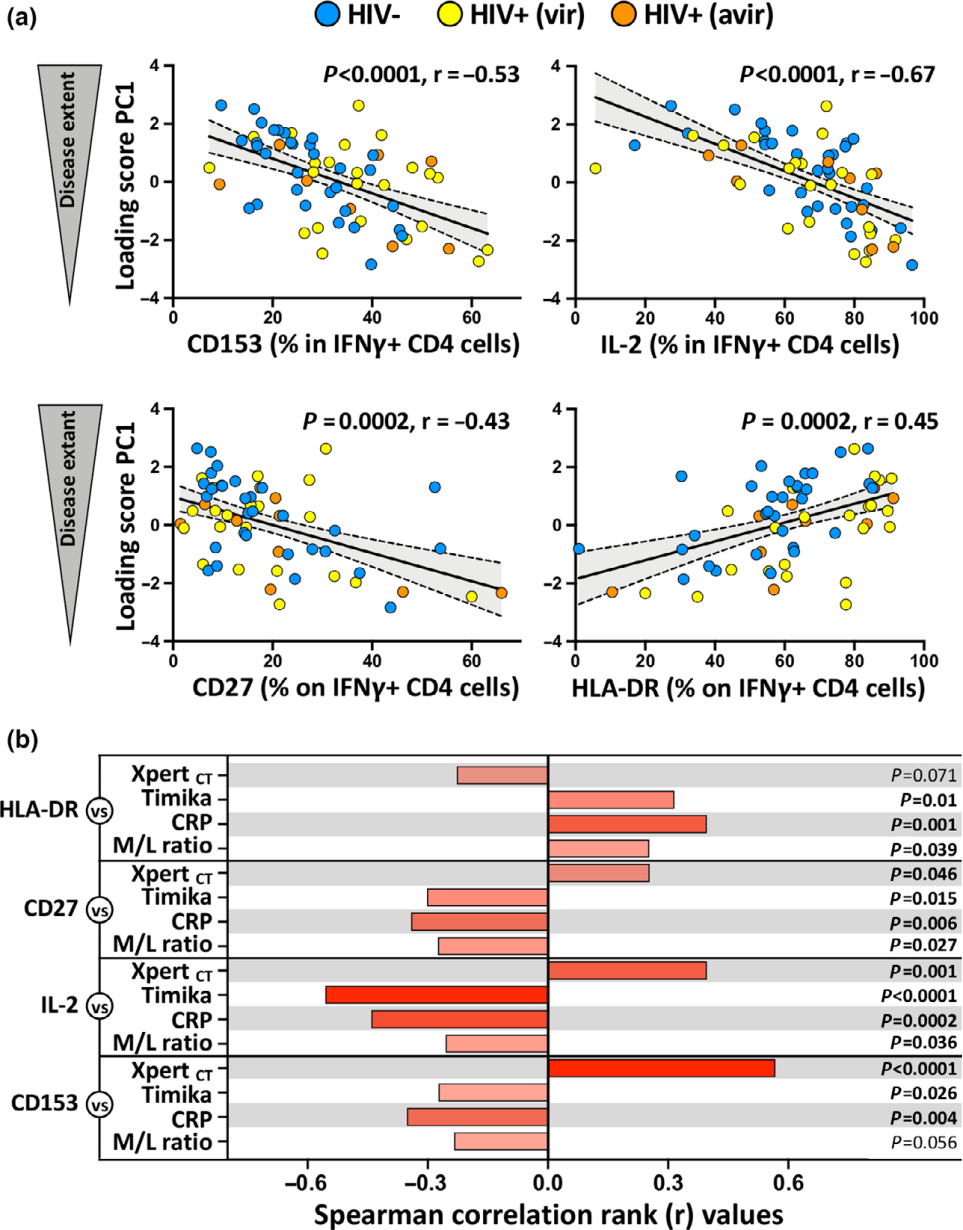
Relationship between Mtb‐specific IFNγ^+^ CD4 T‐cell phenotype and tuberculosis (TB) disease score at baseline. **(a)** Correlation between TB disease score PC1 and CD153, IL‐2, CD27 and HLA‐DR expression by Mtb‐specific IFNγ^+^ CD4 T cells at baseline. active TB (aTB)/HIV^−^ participants are depicted in blue (*n* = 30), aTB/HIV^+^ (viraemic) in yellow (*n* = 10), aTB/HIV^+^ (aviraemic) in orange (*n* = 20). Linear regression and 95% confidence band are depicted. Correlation was tested by a two‐tailed non‐parametric Spearman rank test. **(b)** Pairwise correlations of each phenotypic marker expression by Mtb‐specific IFNγ^+^ CD4 T cells with each clinical parameter. Bars represent the Spearman correlation *r*‐values. The *P*‐values are indicated on the right.

To define the relationship between Mtb300‐specific IFNγ^+^ CD4 T‐cell phenotype and TB disease extent, we next assessed the association of the PC1 loading score (described in Figure 1) with the expression of each measured marker. Strong correlations were observed between the frequency of CD153 (*P* < 0.0001; *r* = −0.53), IL‐2 (*P* < 0.0001; *r* = −0.67), CD27 (*P* = 0.0002; *r* = −0.43) and HLA‐DR (*P* = 0.0002; *r* = 0.45) expression by Mtb300‐specific IFNγ^+^ CD4 T cells and the PC1 loading score, indicative of TB disease extent (Figure 4a). Conversely, no associations were found between the PC1 score of TB disease extent and KLRG1 or MIP‐1β expression (data not shown). Further analyses, using a pairwise comparison approach where each phenotypic marker was compared individually against all the different clinical parameters, show that HLA‐DR and CD27 exhibited the strongest association with plasma CRP concentration (*P* = 0.001, *r* = 0.4, and *P* = 0.006, *r* = −0.34, respectively), and IL‐2 expression exhibited the strongest association with the Timika score (*P* < 0.0001, *r* = −0.55) and CD153 expression with Xpert_ct_ values (*P* < 0.0001, *r* = 0.57; Figure 4b). This suggests that systemic inflammation significantly contributes to Mtb300‐specific IFNγ^+^ CD4 T‐cell differentiation and activation, while high CD153 expression on these cells was associated with lower Mtb bacterial burden.

**Figure 5:**
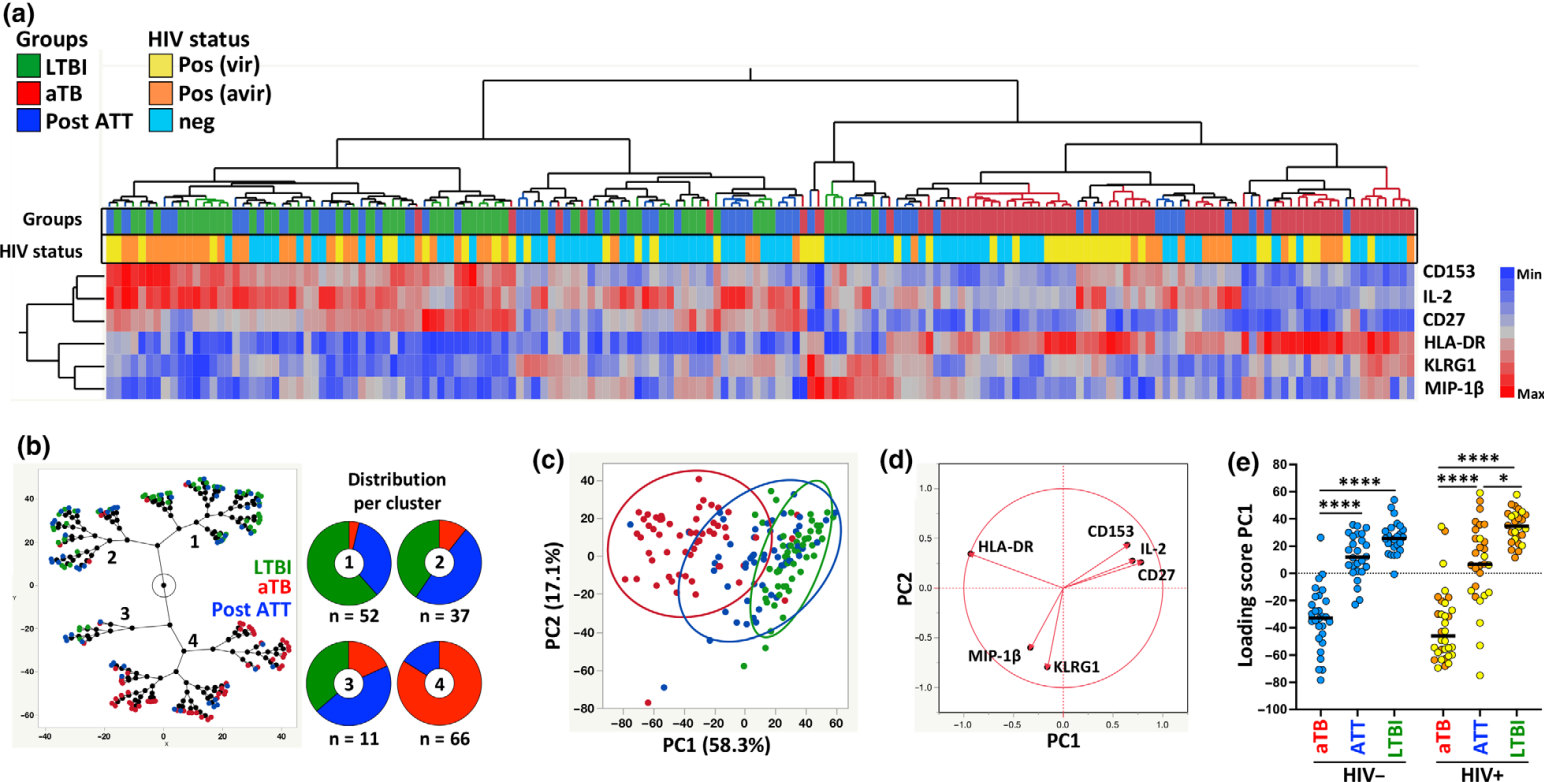
Phenotypic signature of Mtb‐specific IFNγ^+^ CD4^+^ T cells according to tuberculosis (TB) disease activity. **(a)** Non‐supervised two‐way hierarchical cluster analysis (HCA, Ward method) was employed to define whether simultaneous assessment of measured markers (CD153, IL‐2, CD27, HLA‐DR, KLRG1 and MIP‐1β) could group separately individuals based on TB disease activity. TB status (latent tuberculosis infection in green, *n* = 53; active TB [aTB] in red, *n* = 60 and Post‐anti‐tubercular treatment in blue, *n* = 55) and HIV status (HIV‐uninfected (Neg, light blue), viraemic HIV‐infected (Posvir, yellow) and aviraemic HIV‐infected (Posavir, orange)) for each donor are indicated at the bottom of the dendrogram. Data are depicted as a heatmap coloured from minimum to maximum values detected for markers. **(b)** Constellation Plot‐cluster analysis based on all measured markers. Each dot represents a participant and is colour‐coded according to TB disease activity. Each cluster obtained for the HCA is identified by a number. **(c)** Principal component analysis of covariances, derived from the six studied parameters, was used to explain the variance of the data distribution in the cohort. Each dot represents a participant and is colour‐coded according to participant's TB status. The two axes represent principal components 1 (PC1) and 2 (PC2). Their contribution to the total data variance is shown as a percentage. PC3 contributed 9.7% of total variance and is not shown. 90% coverage bivariate normal density ellipses are shown for each group. **(d)** Loading plot showing how each parameter influences PC1 and PC2 values. **(e)** Comparison of PC1 score values between each group. Bars represent medians. Statistical comparisons were calculated using a Kruskal–Wallis test, adjusted for multiple comparisons (Dunn's test; ****P < 0.0001, *P < 0.05).

We next analysed the overall phenotypic signature of Mtb300‐specific IFNγ^+^ CD4 T cells by conducting a hierarchical clustering analysis, where all measured variables were considered simultaneously (Figure 5a). Three main clusters (1, 2 and 4) were identified accounting for 93% of the samples (155/166). Clusters 1 and 2 were characterised by elevated expression of CD153, IL‐2 and CD27 and low expression of HLA‐DR, while cluster 4 presented an opposite profile with low expression of CD153, IL‐2 and CD27 and elevated HLA‐DR. Visualising the distribution of the different study groups (LTBI, aTB pre‐ATT and aTB post ATT) within each cluster using a constellation plot, we observed that cluster 1 was dominated by those who were LTBI and aTB post‐ATT (61% and 35%, respectively), cluster 2 included mostly aTB post‐ATT and LTBI (49% and 40%, respectively), whereas cluster 4 was composed of mostly of aTB pre‐ATT (77%) and 23% of aTB post‐ATT (Figure 5b).

**Figure 6:**
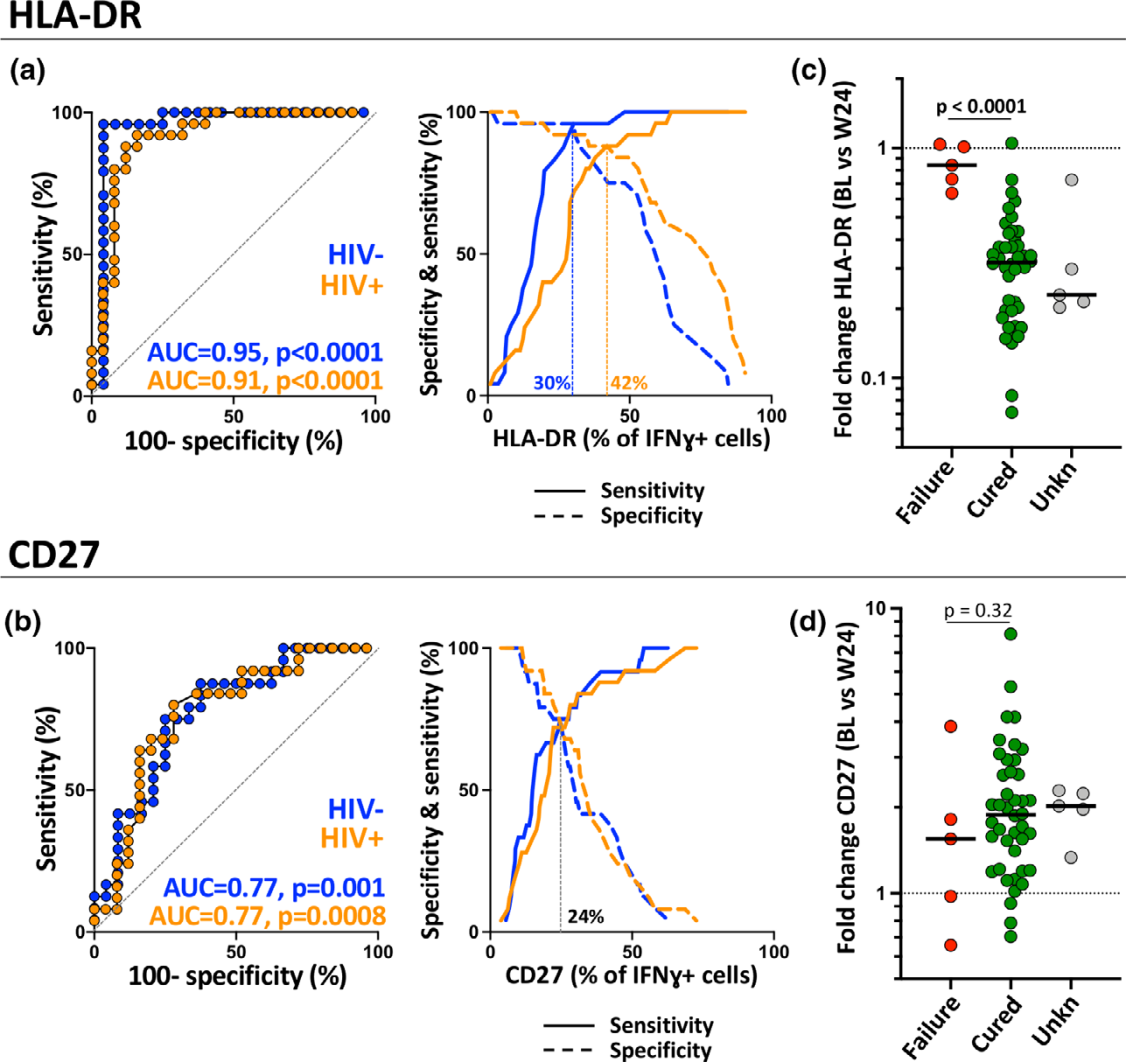
Performance of HLA‐DR and CD27 to discriminate active from treated tuberculosis. **(a, b)** Receiver operating characteristic curves and specificity/sensitivity crossover plots for HLA‐DR and CD27 to discriminate between active from treated tuberculosis (TB) in HIV‐uninfected (blue) and HIV‐infected (orange) individuals. The diagonal dashed line depicts an AUC of 0.5, representing a random test. The dashed vertical line on the crossover plots represents the optimal threshold to distinguish active and successfully treated individuals. **(c, d)** Fold change in HLA‐DR and CD27 expression in Mtb‐specific IFNγ^+^ CD4 T cells between baseline and 24 weeks post‐treatment initiation. Green dots identify patients considerate as cured based on TB treatment guideline (*n* = 42), red dots participants who experience treatment failure or relapse (*n* = 5) and grey dots participants with unknown treatment outcome due to missing sputum culture data (*n* = 5).

To further evaluate the combined power of the measured parameters to discriminate between the three study groups, a PCA was performed. PC1 accounted for 58.3% and PC2 17.1% of the variance (Figure 5c). The PCA showed an almost complete separation between LTBI and aTB pre‐ATT (based on PC1), whereas the profile of aTB post‐ATT group mostly overlapped with that observed for the LTBI group. The corresponding loading plot shows that HLA‐DR, CD27, CD153 and IL‐2 were the main drivers of PC1 variance (Figure 5d). The PC1 scores were then compared between each group stratified according to HIV status. Figure 4e shows that while there was no significant difference between the HIV‐uninfected aTB post‐ATT and LTBI groups, the PC1 score in the aTB post‐ATT group remained significantly lower than that in the LTBI group in HIV‐infected participants. This is indicative of incomplete restoration of the phenotypic profile of Mtb‐specific IFNγ^+^ CD4 T cells in HIV‐1 co‐infected persons, despite completion of the standard ATT regimen. Previous reports have suggested that activation markers such as HLA‐DR and CD38, as well as the expression of the maturation marker CD27 measured individually, may have value in discerning between LTBI, aTB pre‐ATT and aTB post‐ATT.^29^ Receiver operating characteristic curves (Figure 6a, left panel) showed that HLA‐DR discerned with high sensitivity and specificity aTB patients from successfully treated TB patients, irrespective of HIV infection (Area under the curve (AUC) = 0.95, *P* < 0.0001 for HIV‐uninfected and AUC = 0.91, *P* < 0.0001 for HIV‐infected). Moreover, specificity/sensitivity crossover plots showed that successful treatment was associated with HLA‐DR expression on Mtb‐specific CD4 T cell post‐ATT lower than 30% for HIV‐uninfected patients and lower than 42% for HIV‐infected participants (Figure 6a, right panel). CD27, by contrast, displayed much poorer sensitivity and specificity (Figure 6b). Importantly, analysis of the fold change in HLA‐DR expression on IFNγ^+^ CD4 T cells between the pre‐ATT and post‐ATT time points indicated that those who failed treatment or relapsed (*n* = 5) exhibited a significantly lower fold change in HLA‐DR expression than in patients classified as cured (median: 0.84 vs 0.31, respectively, *P* < 0.0001; Figure 6c).

### Relationship between the phenotypic signature of Mtb‐specific IFNγ^+^ CD4 T cells and TB disease extent

**Figure 7:**
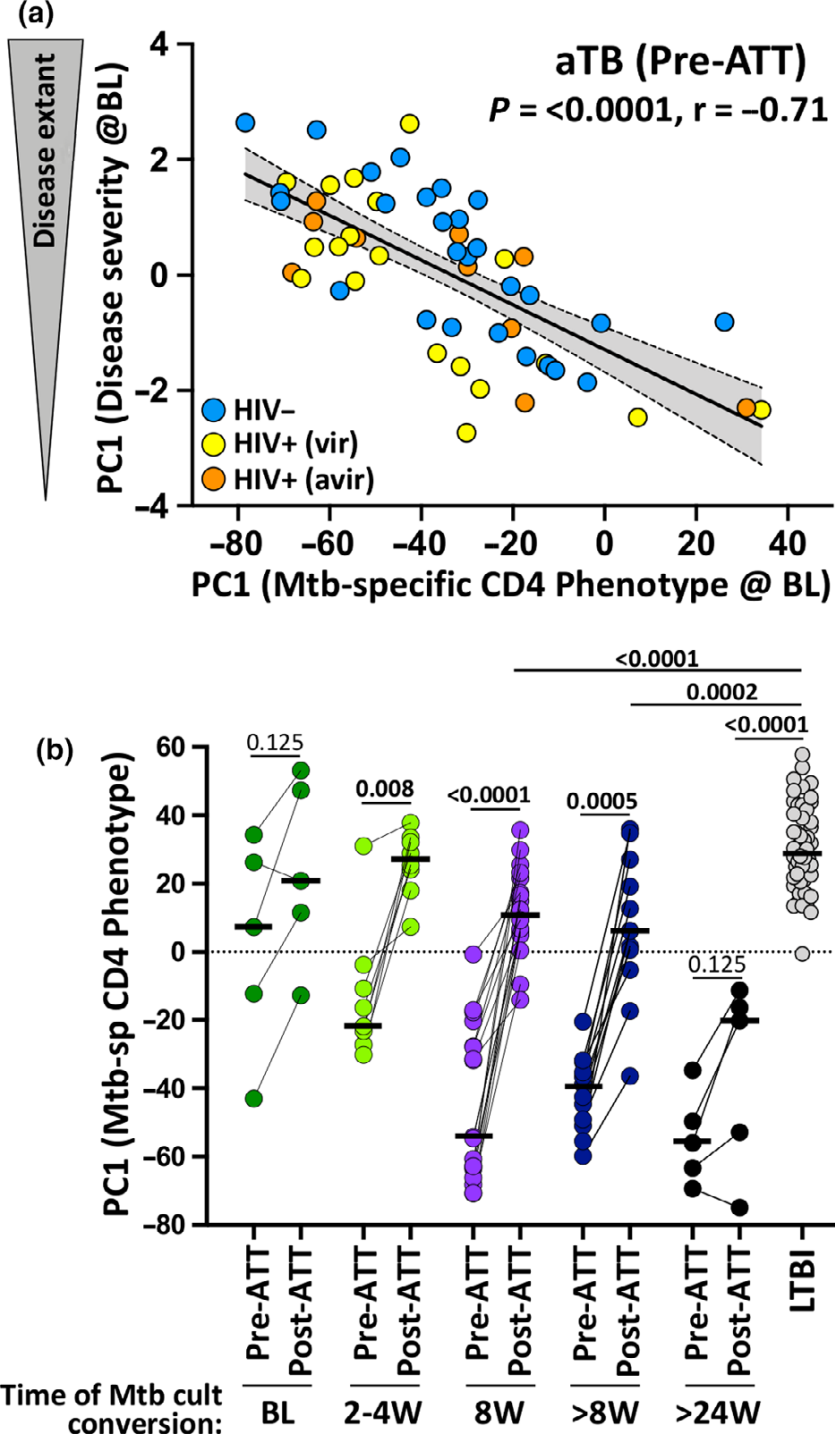
Relationship between Mtb‐specific IFNγ^+^ CD4 T‐cell signature, tuberculosis (TB) disease score and treatment outcome. **(a)** Correlation between TB disease score PC1 and Mtb‐specific CD4 T‐cell signature PC1 at baseline. Active TB (aTB)/HIV^−^ participants are depicted in blue (*n* = 30), aTB/HIV^+^ (viraemic) in yellow (*n* = 10), aTB/HIV^+^ (aviraemic) in orange (*n* = 20). Linear regression and 95% confidence band are depicted. Correlation was tested by a two‐tailed non‐parametric Spearman rank test. **(b)** Comparison of the Mtb‐specific CD4 T‐cell signature PC1 at baseline (Pre‐anti‐tubercular treatment [ATT]) and 24 weeks post‐ATT initiation (Post‐ATT) according to time to Mtb culture conversion. PC1 values obtained from latent tuberculosis infection participants are shown with open circles. Statistical comparisons were performed using a Wilcoxon rank test for paired samples or a Mann–Whitney test for unpaired samples.

Finally, PC1 of the measures of TB disease extent and PC1 of the Mtb‐specific IFNγ^+^ CD4 T cell phenotype at baseline showed strong correlation (*P* < 0.0001; *r* = −0.71), irrespective of HIV infection (*P* < 0.0001; *r* = −0.78 for HIV^−^ and *P* < 0.0001; *r* = −0.67 for HIV^+^, data not shown; Figure 7a), indicating that, unlike the magnitude of Mtb‐specific CD4 response,^32^ the phenotypic characteristics of these cells associate with TB disease activity. Furthermore, only those who showed early sputum culture conversion (e.g. before week 8) had PC1 loading scores for CD4 T‐cell phenotypes at week 24 that were comparable to that of the LTBI group (Figure 7b). Thus, the persistence of a skewed CD4 T‐cell phenotype in those with late Mtb culture conversion (≥ week 8) suggests that there is a lag (~ 16 weeks) between Mtb clearance and the full restoration of Mtb‐specific CD4 T‐cell attributes. Moreover, no significant changes were observed between baseline and treatment completion in the overall phenotypic profile of Mtb‐specific IFNγ^+^ CD4 T cells of those who experience treatment failure or relapse (*P* = 0.125, Figure 7b).

## Discussion

A better understanding of the relationship between the host immune response, TB disease severity and treatment outcome is key to the development of novel TB diagnostic and treatment monitoring tools.^33^ In this study, we defined the profile of Mtb‐specific IFNγ^+^ CD4 T‐cell response in TB patients, analysed its evolution after completion of a standard ATT regimen and evaluated how the host CD4 response associates with clinical TB extent and treatment response. There is great variability in clinical presentation of TB disease.^34^, ^35^, ^36^, ^37^ Here, we show that by combining a number of established clinical variables used in TB diagnosis measuring bacterial burden (Xpert_ct_ value), systemic inflammation (plasma CRP and monocyte/lymphocyte ratio) and radiographic evidence of pulmonary disease extent on chest radiograph (Timika score), we were able to define TB disease extent as a continuum with higher scores associating with delayed culture conversion and unfavorable clinical outcomes (treatment failure and relapse). This finding is in accordance with existing evidence showing that sputum smear grade and/or radiological extent of disease at presentation associates with sputum culture conversion time.^38^, ^39^, ^40^

We then evaluated whether the Mtb‐specific IFNγ^+^ CD4 T‐cell response could inform clinical TB severity. Our data show that while the magnitude of the response was unrelated to TB disease activity, as previously described^16^, ^21^, ^41^, the overall phenotypic profile of these cells strongly associated TB disease extent, irrespective of patient HIV status. When parameters were analysed individually, our data reveal preferential associations between certain markers and clinical features. The extent of lung involvement (measured by Timika score) showed a strong negative association with IL‐2 expression in Mtb‐specific IFNγ^+^ CD4 T cells. HLA‐DR expression on these cells displayed the strongest association with systemic inflammation (defined using plasma CRP concentration) and high bacterial burden (inferred by the Xpert cycle threshold), while CD153 expression on Mtb‐specific IFNγ^+^ CD4 T cells negatively associated with bacterial burden. Two recent publications have highlighted the potential role of CD153 for Mtb protection in murine and non‐human primate Mtb‐infection models^42^ and in a non‐human primate model of intravenous BCG vaccination.^43^ However, at this stage, we cannot infer whether the Mtb‐specific CD4 T‐cell profile is a cause or a consequence of TB disease activity. The mechanisms underlying these observed associations are still unclear, yet our results suggest that the phenotypic attributes of the Mtb‐specific response could be independently determined by distinct clinical features. Overall, our results confirm and expand on previous findings,^21^ showing that the phenotype of Mtb‐specific CD4 T cells associates with TB disease severity.

To date, the gold standard for TB diagnosis relies on detection of Mtb using microscopy, culture or molecular methods.^44^, ^45^ However, these tests present substantial limitations as they require either sputum or a specimen from the site of disease in patients with extrapulmonary TB. It may be particularly difficult to obtain representative sputum samples in children or HIV‐infected persons, where immunosuppression associates with reduced lung cavity formation.^46^ Additionally, in those with extrapulmonary TB, it is frequently difficult or impossible to obtain a specimen from the site of disease as this requires invasive sampling procedures, necessitating heavy reliance on clinical diagnosis and empiric treatment. Our study adds to the mounting evidence that simple whole blood‐based assays measuring particular Mtb‐specific CD4 T‐cell maturation and activation markers can be applied not only in the diagnosis of TB,^15^, ^18^, ^19^, ^20^ but also in the assessment of TB disease severity at presentation which is indicative of prognosis, irrespective of HIV co‐infection.

Current sputum culture and smear‐based assessment of treatment response to standard TB treatment regimens and investigational new drugs also have significant limitations.^47^ While sputum culture conversion at 2 months of ATT correlates with clinical cure and lower rates of relapse,^48^ it failed as single measure to determine whether shortened, 4‐month ATT regimens would be successful with significantly more frequent relapse being observed in those treated for only 4 months compared to conventional 6‐month regimens.^49^ Moreover, lack of specificity limits the use of sputum Xpert to monitor the response to ATT.^9^ We thus investigated whether the phenotypic attributes of Mtb‐specific IFNγ^+^ CD4 T cells could be relevant to monitor treatment response. Our results showed that, upon ATT completion, the phenotypic profile of Mtb‐specific CD4 cells reverts to profiles that were comparable to those of LTBI participants. However, complete normalisation was not observed in all participants, most notably in those co‐infected with HIV. Furthermore, all participants with incomplete restoration of their Mtb‐specific CD4 T‐cell profile post‐ATT (e.g. with phenotypic scores significantly lower than that of the LTBI groups) exhibited delayed sputum culture conversion (at or beyond week 8 of ATT). One might hypothesise that those exhibiting phenotypic scores in the ranges of untreated aTB at the end of their ATT regimen may be at particular risk for poor treatment outcomes (treatment failure/relapse) and recurrence, and although further studies would be required to confirm this, we did find that this was indeed the case in the a small number of participants (*n* = 5) experiencing treatment failure/relapse in our cohort.

Translation of whole blood assay‐based approaches into diagnostic and treatment monitoring tools requires use of simplified flow cytometry panels. Upon focusing our analyses solely on HLA‐DR expression, our data concur with previous findings from our group and others that a 4‐colour panel (including CD3, CD4, IFN‐γ and HLA‐DR) could discriminate latent from aTB^15^, ^18^, ^19^, ^20^ and could also be useful to monitor the response to treatment, as recently suggested by Ahmed *et al*.^29^ Here, we show for the first time that this approach: (1) could identify those at highest risk of poor treatment outcomes, (2) could gauge the extent of TB disease severity and (3) importantly, exhibit comparable performance in HIV‐infected persons, who are at highest risk of TB disease. While flow cytometry may appear complex, its usage in clinical tests is becoming more common with the availability of compact flow cytometers. Flow cytometry‐based assays, such as CD4 testing, are already in use in programmatic settings globally, including in resource‐constrained settings. The translation of a whole blood assay into a point of care product is thus conceivable for clinical reference laboratories. Further development would however be required (e.g. using tubes containing lyophilised reagents for cell stimulation, pre‐mixed antibody cocktails for cell staining and automated gating for flow analyses) to ensure commercial scalability.

The study was limited by the fact that it was conducted on ambulant outpatients attending a combined HIV‐TB clinic, thus potentially biasing especially the HIV‐1 co‐infected population towards a less severely diseased phenotype. However, CRP and M/L ratios were comparable between HIV‐1‐infected and HIV‐1‐uninfected aTB groups at baseline, indicating comparable levels of systemic inflammation induced by TB infection in these two groups. Sputum culture was limited to pre‐determined study visit dates and thus could not be ascertained at more regular intervals. There were missing sputum culture results in a number of patients due to unattended study visits and culture contamination; however, these were in the minority. It also remains to be defined whether Mtb‐specific CD4 T‐cell phenotyping could be used for early identification of and targeted intervention in those at highest risk for poor treatment outcomes and whether the normalisation of these markers is indicative of relapse‐free cure.

## Methods

### Study population

Participants were recruited from the Ubuntu Clinic, Site B, Khayelitsha (Cape Town, South Africa) between March 2017 and December 2018. All participants were adults (age ≥ 18 years) and provided written informed consent. The study was approved by the University of Cape Town Human Research Ethics Committee (HREC 050/2015) and was conducted under DMID protocol no. 15‐0047. Those in the aTB group (*n* = 95) all tested sputum Xpert MTB/RIF (Xpert, Cepheid, Sunnyvale, CA, USA) positive and had clinical symptoms and/or radiographic evidence of TB. All aTB cases were drug sensitive and had received no more than one dose of ATT at the time of baseline blood sampling. The latent TB healthy control group (*n* = 66) were all asymptomatic, had a positive IFN‐γ release assay (IGRA, QuantiFERON®‐TB Gold In‐Tube, Qiagen, Hilden, Germany), tested sputum Xpert MTB/RIF negative and exhibited no clinical evidence of aTB. Clinical characteristics of the study participants are shown in Table 1. Sputum Xpert MTB/RIF, sputum culture for Mtb, CD4 count, HIV VL, differential full blood count and CRP tests were performed by the South African National Health Laboratory Services. All participants were followed up over the duration of their ATT. Sputum culture was performed at baseline, week 2, week 4 and week 8. Sputum culture was repeated for those where treatment failure or relapse was suspected. Time of sputum culture conversion was defined as the timepoint where the sputum culture result changed from a previous positive result to negative and maintained negative status for the duration of follow‐up sampling. Details of those who tested sputum culture negative at baseline are included in Supplementary table 1.

### Timika scoring of chest radiographs

Chest radiographs from the enrolment visit of those with aTB were scored by the study clinician using the Timika score.^50^ Previously published standardised user guidelines were strictly adhered to.^38^ Briefly, posteroanterior chest radiographs were assessed for the total percentage of the lung fields affected by known features of active pulmonary TB. A value of 40 was added to the overall percentage affected lung where at least one cavity ≥ 1 cm could be identified.

### Blood collection and whole blood assay

Blood was collected in sodium heparin tubes and processed within 3 h of collection. The whole blood assay was adapted from the protocol described by Hanekom *et al*.^51^ Briefly, 0.5 mL of whole blood was stimulated with a pool of 300 Mtb‐derived peptides (Mtb300, 2 µg mL^−1^)^52^ at 37°C for 5 h in the presence of the co‐stimulatory antibodies, anti‐CD28 and anti‐CD49d (1 μg mL^−1^ each; BD Biosciences, San Jose, CA, USA) and Brefeldin‐A (10 µg mL^−1^; Sigma‐Aldrich, St Louis, MO, USA). Unstimulated cells were incubated with co‐stimulatory antibodies and Brefeldin‐A only. Red blood cells were then lysed in a 150 mm NH_4_Cl, 10 mm KHCO_3_ and 1 mm Na_4_EDTA solution. Cells were stained with a Live/Dead Near‐InfraRed dye (Invitrogen, Carlsbad, CA, USA) and then fixed using a Transcription Factor Fixation buffer (eBioscience, San Diego, CA, USA), cryopreserved in freezing media (50% foetal bovine serum, 40% RPMI and 10% dimethyl sulfoxide) and stored in liquid nitrogen until use.

### Cell staining and flow cytometry

Cryopreserved cells were thawed, washed and permeabilised with a Transcription Factor perm/wash buffer (eBioscience). Cells were then stained at room temperature for 45 min with the following antibodies: CD3 BV650 (OKT3; BioLegend, San Diego, CA, USA), CD4 BV785 (OKT4; BioLegend), CD8 BV510 (RPA‐T8; BioLegend), CD27 PE‐Cy5 (1A4CD27; Beckman Coulter, Brea), HLA‐DR BV605 (L243; BioLegend), Killer cell Lectin‐like Receptor G1 (KLRG1) PerCP‐eFluor 710 (13F12F2; eBioscience), IFN‐γ BV711 (4S.B3; BioLegend), TNF‐α eFluor 450 (Mab11; BioLegend eBioscience), IL‐2 PE/Dazzle (MQ1‐17H12, BioLegend eBioscience) MIP‐1β Alexa Fluor 488 (#24006; R&D systems, Minneapolis, MN, USA) and CD153 (R&D116614; R&D systems). Samples were acquired on a BD LSR‐II and analysed using FlowJo (v9.9.6, FlowJo LCC, Ashland, OR, USA). A positive cytokine response was defined as at least twice the background of unstimulated cells. To define the phenotype of Mtb300‐specific CD4 T cells, a cut‐off of 30 events was used.

### Statistical analyses

Graphical representations were done using JMP (v14.0.0; SAS Institute, Cary, NC, USA) and Prism (v8.4.3; GraphPad Software Inc, San Diego, CA, USA). Statistical tests were performed in Prism. Non‐parametric tests were used for all comparisons. The Kruskal–Wallis test with Dunn's multiple comparison test was used for multiple comparisons and the Mann–Whitney and Wilcoxon matched pairs test for unmatched and paired samples, respectively.

## Author Contribution

**Catherine Riou:** Conceptualization; Data curation; Formal analysis; Funding acquisition; Investigation; Methodology; Writing‐original draft. **Elsa Du Bruyn:** Conceptualization; Data curation; Formal analysis; Investigation; Methodology; Writing‐original draft. **Sruzive Ruzive:** Methodology. **Rene T Goliath:** Project administration. **Cecilia S Lindestam Arlehamn:** Resources. **Alex Sette:** Resources. **Alan Sher:** Conceptualization; Funding acquisition; Writing‐review & editing. **Daniel L Barber:** Conceptualization; Writing‐review & editing. **Robert J Wilkinson:** Conceptualization; Funding acquisition; Writing‐review & editing.

## Conflict of interest

The authors declare no conflict of interest.

## Supporting information

Supplementary figure 1

Supplementary figure 2

Supplementary figure 3

Supplementary table 1
