# Supplementary figures and images for: Disease extent and anti‐tubercular treatment response correlates with Mycobacterium tuberculosis‐specific CD4 T‐cell phenotype regardless of HIV‐1 status
Source: Clin Transl Immunology. 2020 Sep 28;9(9):e1176. doi: 10.1002/cti2.1176 (PMC7520805; doi:10.1002/cti2.1176)

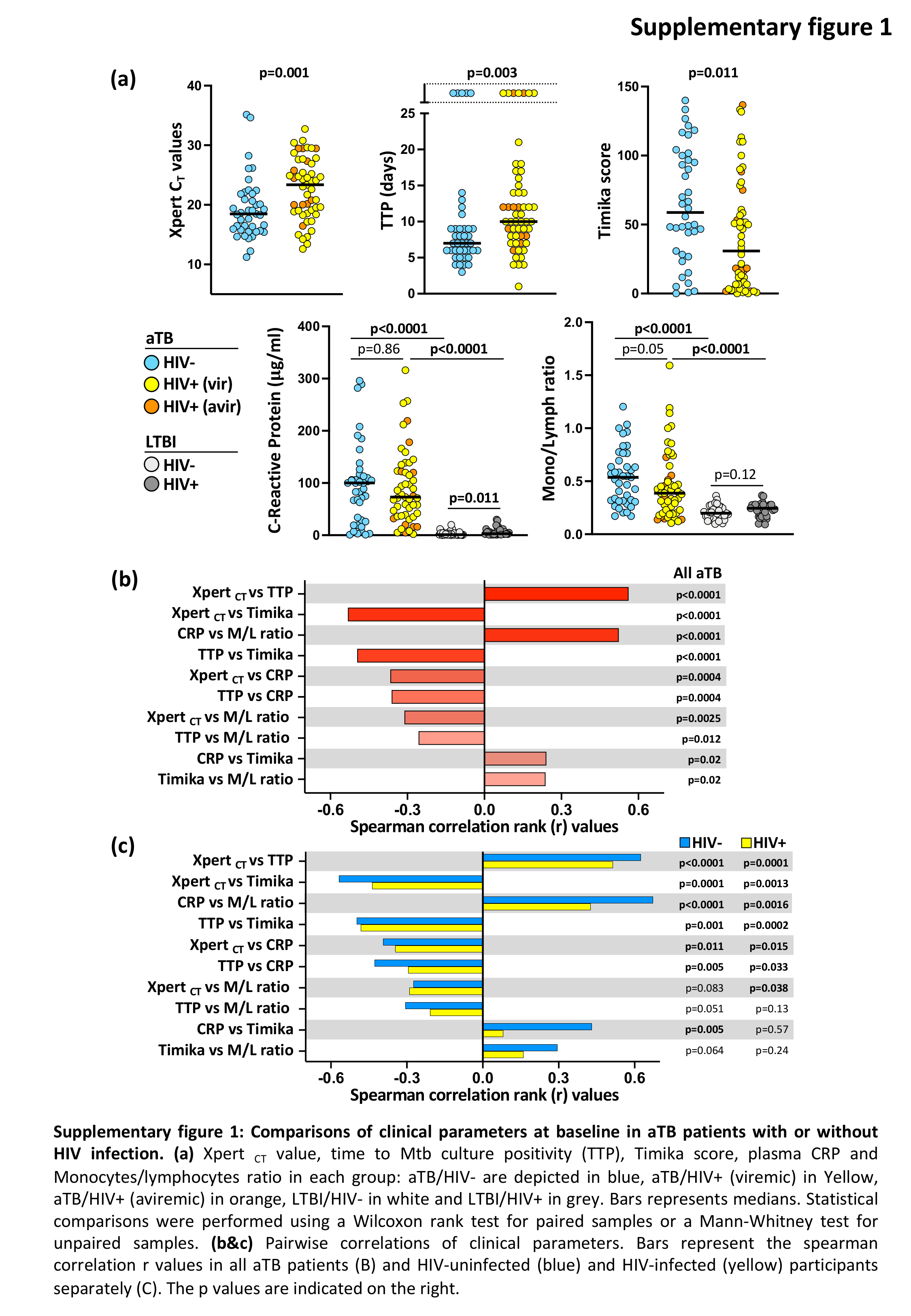

Supplement: Supplementary file 1 — Supplementary figure 1 [file CTI2-9-e1176-s001.tif]

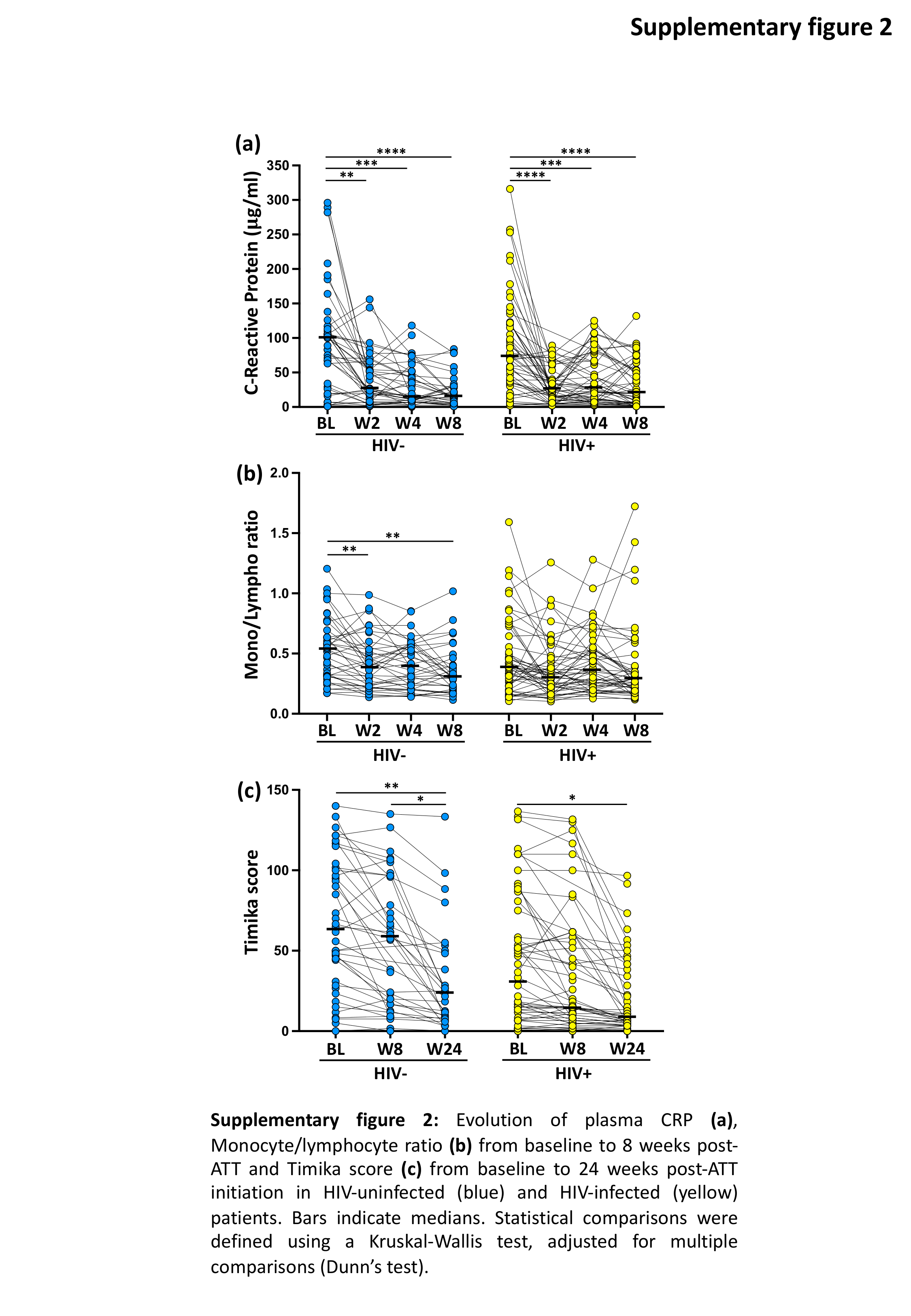

Supplement: Supplementary file 2 — Supplementary figure 2 [file CTI2-9-e1176-s002.tif]

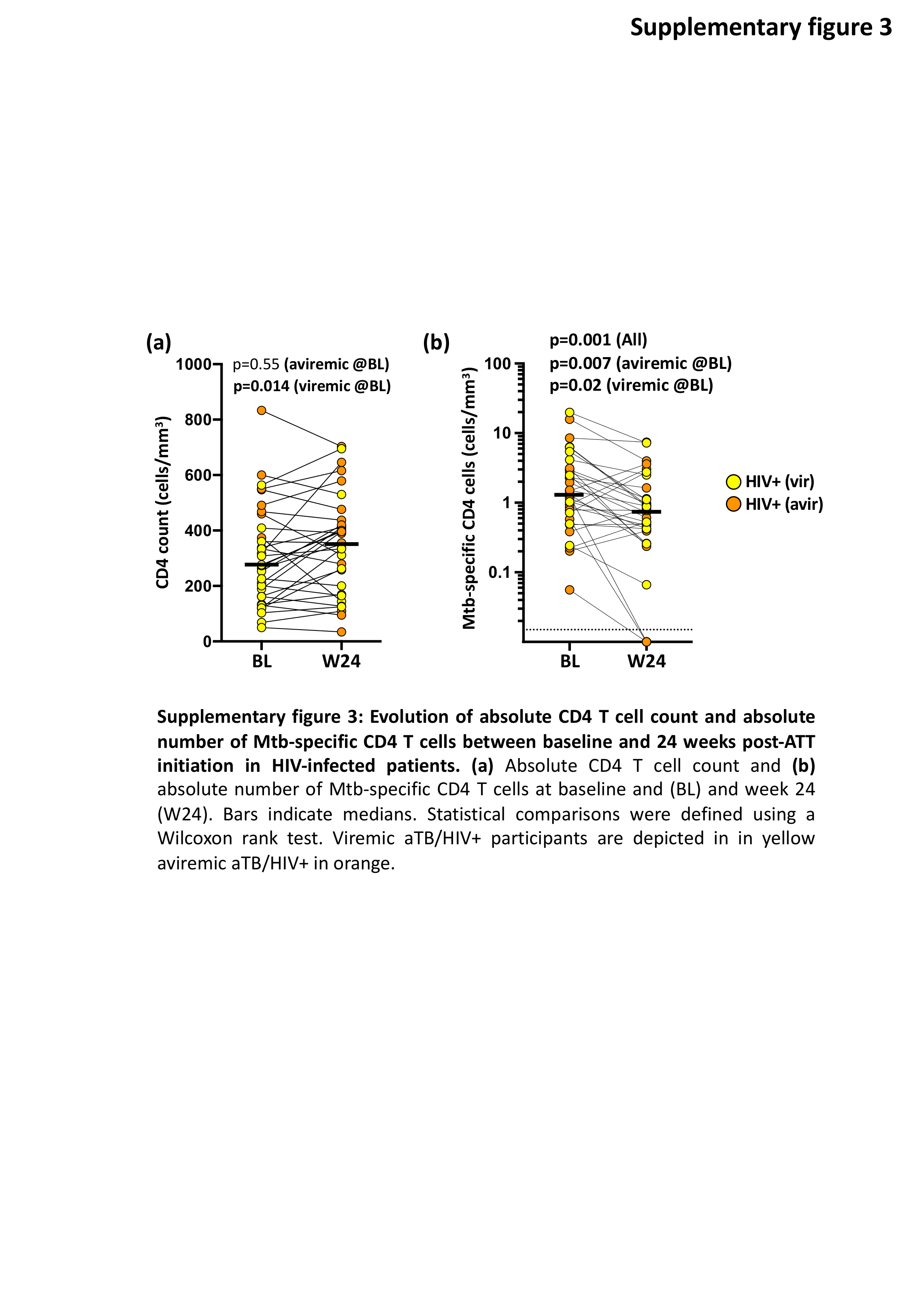

Supplement: Supplementary file 3 — Supplementary figure 3 [file CTI2-9-e1176-s003.tif]
