# Supplementary material for: Disease extent and anti‐tubercular treatment response correlates with Mycobacterium tuberculosis‐specific CD4 T‐cell phenotype regardless of HIV‐1 status
Source: Clin Transl Immunology. 2020 Sep 28;9(9):e1176. doi: 10.1002/cti2.1176 (PMC7520805; doi:10.1002/cti2.1176)
Supplement: Supplementary file 4 — Supplementary table 1 [file CTI2-9-e1176-s004.docx]

**Supplementary table 1**

| **PID** | **Sex** | **Age** | **HIV status** | **CD4 (cells/mm^3^)** | **VL**  **(mRNA copies/ml)** | **ART usage** | **Loss of weight** | **Fever** | **Night sweats** | **Cough** | **Chest pain** | **Shortness of breath** | **Haemoptysis** | **Xpert _CT_ value** | **Timika score** | **M/L ratio** | **CRP (µg/ml)** | **ESR (mm/h)** | **CT scan findings** |
| --- | --- | --- | --- | --- | --- | --- | --- | --- | --- | --- | --- | --- | --- | --- | --- | --- | --- | --- | --- |
| 1084 | F | 31 | Neg | nd | na | na |  |  |  | **✓** | **✓** | **✓** | **✓** | 18.7 | 18.3 | 0.29 | 67 | 76 | Segmental reticular shadowing, left-sided pleural effusion |
| 1077 | M | 41 | Neg | nd | na | na | **✓** | **✓** | **✓** |  | **✓** | **✓** |  | 19.2 | 45 | 0.31 | 19 | 94 | Nodular and confluent opacification, cavity, bronchiectasis, pleural thickening and mediastinal and hilar lymphadenopathy |
| 1012 | M | 33 | Neg | nd | na | na | **✓** | **✓** | **✓** |  | **✓** | **✓** |  | 28.2 | 11.7 | 0.58 | 7 | 26 | Fibrotic changes, bronchiectasis, tree-in-bud nodularity and atelectasis, pleural thickening and hilar lymphadenopathy |
| 1088 | M | 59 | Neg | nd | na | na | **✓** | **✓** | **✓** |  |  | **✓** | **✓** | 34.6 | 7.5 | 0.31 | 1 | 52 | nd |
| 1037 | F | 42 | Neg | nd | na | na | **✓** | **✓** | **✓** |  | **✓** | **✓** |  | 35.1 | 8.3 | 0.32 | 1 | 7 | Fibrosis, peribronchovascular thickening, alveolar nodularity, bronchiectasis and cystic changes |
| 1005 | F | 24 | Pos | 374 | <20 | yes | **✓** | **✓** |  | **✓** | **✓** |  |  | 20.0 | 1.7 | 0.15 | 5 | 30 | Alveolar nodularity, bronchial wall thickening and cicatrization, bronchiectasis and alveolar nodularity |
| 1002 | F | 41 | Pos | 460 | <20 | yes | **✓** | **✓** |  | **✓** |  | **✓** |  | 27.3 | 1.7 | 0.36 | nd | >140 | nd |
| 1027 | M | 47 | Pos | 261 | 284 | yes | **✓** |  |  |  |  |  |  | 29.6 | 15 | 0.28 | 30 | 100 | Confluent nodularity, consolidation, fibrosis, apical bullous changes, fibrocavitatory changes and cicatrizing bronchiectasis |
| 1013 | F | 33 | Pos | 360 | 64,437 | defaulted |  |  |  |  |  |  |  | 30.8 | 0.8 | 0.33 | 8 | 127 | RUL peribronchial infiltrate |
| 1003 | M | 31 | Pos | 338 | 77,741 | no | **✓** | **✓** | **✓** | **✓** |  |  |  | 30.4 | 0.8 | 0.26 | 2 | 72 | Nodular infiltrates, diffuse bronchial wall thickening and early bronchiectasis |

**Supplementary table 1. Clinical characteristics of study participants with negative sputum culture at enrolment (n = 10).**

Definition of abbreviations: PID = Patient identification number; F = female; M = male; VL = HIV-1 viral load; ART = anti-retroviral treatment;

Xpert C_T_ = Xpert MTB/RIF cycle threshold; M/L ratio = monocyte/lymphocyte ratio; CRP = C-reactive protein; ESR = erythrocyte sedimentation rate;

nd = not done; na = not applicable. Xpert cycle threshold value for each participant was defined as the average value of the 5 probes used in the assay.
